# Knowledge and understanding of health insurance: challenges and remedies

**DOI:** 10.1186/s13584-017-0163-2

**Published:** 2017-07-13

**Authors:** Andrew J. Barnes, Yaniv Hanoch

**Affiliations:** 1 Health Behavior and Policy, School of Medicine, Virginia Commonwealth University, 830 E Main St, 9th Floor, Richmond, Virginia, 23219 USA; 2 School of Psychology, Plymouth University, B204, Portland Square, Drake Circus, Plymouth, Devon PL4 8AA UK

**Keywords:** Choice architecture, Health insurance, Literacy, Numeracy

## Abstract

As coverage is expanded in health systems that rely on consumers to choose health insurance plans that best meet their needs, interest in whether consumers possess sufficient understanding of health insurance to make good coverage decisions is growing. The recent IJHPR article by Green and colleagues—examining understanding of supplementary health insurance (SHI) among Israeli consumers—provides an important and timely answer to the above question. Indeed, their study addresses similar problems to the ones identified in the US health care market, with two notable findings. First, they show that overall—regardless of demographic variables—there are low levels of knowledge about SHI, which the literature has come to refer to more broadly as “health insurance literacy.” Second, they find a significant disparity in health insurance literacy between different SES groups, where Jews were significantly more knowledgeable about SHI compared to their Arab counterparts.

The authors’ findings are consistent with a growing body of literature from the U.S. and elsewhere, including our own, presenting evidence that consumers struggle with understanding and using health insurance. Studies in the U.S. have also found that difficulties are generally more acute for populations considered the most vulnerable and consequently most in need of adequate and affordable health insurance coverage.

The authors’ findings call attention to the need to tailor communication strategies aimed at mitigating health insurance literacy and, ultimately, access and outcomes disparities among vulnerable populations in Israel and elsewhere. It also raises the importance of creating insurance choice environments in health systems relying on consumers to make coverage decisions that facilitate the decision process by using “choice architecture” to, among other things, simplify plan information and highlight meaningful differences between coverage options.

## Main text

A major policy drama is taking place in the US where the government is in the process of deciding whether to repeal and replace the ACA (better known as Obamacare). The program, among other things, offers health coverage for millions of Americans who have never held or purchased health insurance in their lives and is the reason for the historically high rates of insurance coverage in the US currently. Despite these successes in coverage expansion, many consumers—especially minorities and low SES individuals—have limited knowledge about the nature and terminology of health insurance [1], with growing indication that consumers are having difficulty in purchasing insurance plans that offer them adequate risk protection [2]. Obamacare, however, is not unique in facing this problem. An earlier US coverage expansion, known as Medicare part D, which offers standalone prescription drug coverage to (mainly) older adults, has exposed similar patterns. Indeed, empirical studies and secondary data analysis have repeatedly shown that beneficiaries do not have full command of the program and often, for example, focus on premiums rather than total expected cost leading to higher overall costs [3].

Much of our knowledge about consumers’ understanding of and decisions about health insurance is based on studies from the US health care market. One might wonder, therefore, if these findings are solely endemic to the US, or whether they can be generalized to other countries and populations.

The paper by Green and colleagues—examining understanding of supplementary health insurance (SHI) among Israeli consumers—provides important and timely information about the experience of consumers outside the US [4]. Indeed, their study addresses similar problems to the ones identified in the US health care market, with two notable findings. First, they show that overall—regardless of demographic variables—there are low levels of knowledge about SHI, which the literature has come to refer to more broadly as “health insurance literacy.” Indeed, Green et al. report that less than 50% of the participants could answer questions correctly about the various services covered by SHI (see [4], Table 2), and about a third of the sample indicated that they have never even examined what coverage the SHI offers. Their findings, it might be argued, are slightly more alarming than those typically reported among US participants, as the coverage rates of SHI among participants is rather high (about 77% of the sample). That is, participants’ poor knowledge about SHI did not stem from lack of experience, but from variables that are yet to be investigated.

Green et al.’s second main result shows the existence of a significant disparity in health insurance literacy between different SES groups, where Jews were more knowledgeable about SHI compared to their Arab counterparts. The gap persisted even after controlling for sociodemographic descriptors that might confound the relationship between ethnicity and health insurance literacy (e.g., education, socioeconomic status, SHI ownership), suggesting a critical disconnect between Israelis' perceptions of what services SHI covers and what services SHI actually covers.

The authors’ findings have empirical support from a growing body of literature, including our own, presenting consistent evidence that consumers struggle with understanding and using health insurance. Studies in the US have found that these difficulties are generally more acute for populations considered the most vulnerable and consequently most in need of adequate and affordable health insurance coverage. Health systems, like Israel’s and many others, which rely heavily on consumers’ ability to choose and use coverage, should be concerned that the populace has sufficient levels of health insurance literacy to understand the structure of health benefits and basic cost-sharing concepts well enough to make effective choices [5].

To understand the pervasive lack of health insurance literacy among many populations and the implications of this deficit on consumers’ ability to choose and use health insurance, consider again the US, where most of our research on this topic has been conducted. More than half of the US adult population lacks the facility with mathematics essential to understand health insurance information [6].^1^ Previous studies have shown that insured people do not understand key insurance terms, risk, and the likely out-of-pocket costs when they experience an illness, nor do they understand what is and is not covered by their insurance plans [7–9].

Limited understanding of health insurance is particularly acute among low-income and otherwise disadvantaged populations [2, 8–10]. Several studies demonstrate that poor health insurance literacy results in people making unambiguously bad choices for themselves, leading to excess medical spending, with older and lower income individuals worst off [11, 12]. Similarly, Green et al. emphasize that Arab populations in Israel, whom they showed to have lower health insurance literacy, tend to be in poorer health and have lower income, less education, and worse access to health care when compared to Jews living in Israel, contributing to the “inequality in the (Israeli) health system” [4]. Importantly, Green et al.’s results provide preliminary evidence supporting ethnicity as a unique marker for low health insurance literacy among Israelis even after controlling for socioeconomic status, education, and access to health care.

While the work of Green et al. makes an important contribution to the literature, the next phase of this line of inquiry should, we believe, focus on addressing low levels of health insurance literacy generally and among more vulnerable populations specifically. Needless to say, no magical formula exists that can easily solve this complex problem. However, our own research and that of others has highlighted three possible avenues. First, policymakers and supplementary health insurance funds should ensure that SHI information (e.g., leaflets) is presented and communicated in a range of languages and in a simplified way (e.g., avoiding technical terms), such that individuals from all sections of the population can read and understand it. SHI funds, for example, can imitate the way health care providers have tailored information to effectively communicate with patients and developed a shared decision-making model [13]. Second, SHI funds can improve the SHI decision environment. Better known as choice architecture, a growing body of research—largely inspired by the emerging field of behavioral economics—has devoted much effort and time to examining ways to improve the decision environment in which consumers operate. Some options to do so that payers can utilize include: reduce the number of SHI options consumers face, present choices in order of price and/or quality, create defaults, use symbolic representation, and standardize coverage options [14]. Third, SHI funds can coordinate with Arab community groups to target outreach and tailor SHI enrollment and education campaigns to improve how these populations understand and use health care coverage. These are some promising mechanisms that have been identified previously. Future research would need to evaluate their feasibility and appropriateness to the SHI market in Israel, and possibly develop novel ways to address the problem.

## Conclusions

When there is a mismatch between health care needs and plan choices resulting from poor health insurance literacy, consumers may not have adequate risk protection to cover their expected health care needs or they may purchase unnecessary coverage. Green and colleagues’ important findings add to a growing literature on health insurance literacy, most of which concludes that consumers do not understand key health insurance terms and have difficulty aligning what they want in an insurance plan with what they choose [15]. The authors’ findings call attention to the need to tailor communication strategies aimed at mitigating health insurance literacy and, ultimately, access and outcomes disparities among vulnerable populations in Israel and elsewhere. It also highlights the importance of creating choice environments that facilitate the decision process, referred to as “choice architecture,” in health systems relying on consumers to make coverage decisions. Indeed, our own work has revealed that participants with both high and low health insurance literacy benefit from simplifying coverage choices by equal amounts. However the magnitude of this effect represented a larger relative increase among participants with lower health insurance literacy given the disadvantage with which these participants came to the coverage choice environment [16].

## Abbreviations

SHI: Supplementary health insurance
US: United States

## Footnotes

1 Numeracy and literacy levels among Israeli adults are below OECD average (see http://www.oecd.org/skills/piaac/Skills-Matter-Israel.pdf). As such, there is little reason to believe that the results from the US would be dramatically different.
